# Participatory development and proof-of-concept of an intersectionality-informed art-based group intervention for BIPoC girls

**DOI:** 10.1038/s41598-026-40521-7

**Published:** 2026-02-17

**Authors:** Susanne Birnkammer, Rayan El-Haj-Mohamad, Claudia Calvano

**Affiliations:** 1 Division of Clinical Child and Adolescent Psychology and Psychotherapy, Department of Education and Psychology, Freie Universität Berlin, Berlin, Germany; 2 Division of Clinical Psychological Interventions, Department of Education and Psychology, Freie Universität Berlin, Berlin, Germany; 3 Partner Site Berlin-Potsdam, German Center for Mental Health (DZPG), Berlin, Germany

**Keywords:** Health care, Psychology, Psychology

## Abstract

While racism and its negative mental health impact are widespread problems for BIPoC (Black, Indigenous, and People of Color) girls in Germany, intersectionality-informed psychological interventions remain unavailable. To address this gap, this study piloted an youth-informed art-based group intervention designed to strengthen girls’ internal resources (e.g., self-worth, self-efficacy) and reduce mental health symptoms. Conducted in three phases, it involved: (1) a focus group with BIPoC girls (*n* = 8) to explore needs and preferences; (2) pilot testing of a self-reflective weekly journal to assess its feasibility; and (3) a six-week art-based group intervention with BIPoC girls (*n* = 7, aged 14–16) to evaluate feasibility and acceptability using a mixed method approach. Feasibility was supported by high attendance (71% attended ≥ 4 sessions) and positive session ratings (92.9% very good/good); acceptability was reflected in high satisfaction, perceived emotional safety, cultural resonance, and valuing of creative activities, with 75% reporting improved well-being after sessions. Preliminary outcomes showed small descriptive increases in self-efficacy and self-worth, and post-session gains in self-satisfaction. The journal was viewed as a potentially useful self-regulatory tool but had low uptake. These findings provide valuable directions for shaping intersectionality-informed, creative group interventions for BIPoC girls in Germany.

**Supplementary Information:**

The online version contains supplementary material available at 10.1038/s41598-026-40521-7.

## Introduction

Racial discrimination remains a persistent and growing concern across Europe. According to the EU-MIDIS II survey conducted across EU member states, 39% of respondents from racialized populations reported experiences of racial discrimination within the past five years, and 24% reported such experiences in the preceding 12 months^1^. These findings reflect long-standing concerns about the persistence and recent increase of racial discrimination across Europe^2^, with children and adolescents being particularly affected^3–5^.

In its most recent EU reporting, Germany was identified as one of the countries with the highest rates of discrimination, racist violence, and structural racism^6^. Among racialized youth in Germany, 73% of those aged 14–24 report having experienced racism, making them the most affected age group^4^. Such experiences occur across different daily contexts, including schools, peer interactions, and public spaces^7–9^.

Adolescence represents a particularly sensitive developmental period due to its central role in identity formation and emotional development^10–12^. Sequential micro-traumatization describes how repeated racialized encounters can accumulate and result in long-term traumatic effects^13,14^. Cumulative exposure to racial discrimination has been shown to impair emotional regulation, heighten stress sensitivity, and contribute to depression, post-traumatic stress disorder (PTSD) symptoms, substance use, and suicide risk^8,15–17^.

Intersectionality theory^18^ underscores how overlapping systems of oppression compound risk and intensify mental health inequities^19,20^. Racialized girls, including Black, Indigenous, and other girls of Color (BIPoC), occupy the intersection of racialized and gendered identities and face distinct challenges^2,21^. They are exposed to gendered racial invalidation and stereotypes that undermine their social value^22,23^. For example, harmful cultural tropes such as the stereotype of the “angry Black girl” can contribute to social isolation, identity suppression, and negative academic outcomes^24,25^. These layered experiences are associated with a broad range of adverse mental health outcomes, including internalizing and externalizing symptoms and trauma-related distress^20,26^.

Despite the urgent need for effective psychological support, individually focused Western psychotherapeutic approaches often fail to incorporate socio-ecological perspectives or the societal contexts of discrimination, and therefore do not adequately address the specific needs of youth with intersectional identities^27,28^. These approaches typically rely on spoken dialogue and standardized, manualized protocols, whose evidence base was largely established in Western, White populations^29^. This limits their alignment with the cultural contexts, lived experiences, and preferred modes of expression of BIPoC adolescents^30,31^. In addition, BIPoC youth may encounter racial microaggressions within therapeutic settings^32^, which can reinforce distrust and retraumatization. In Germany, this lack of culturally responsive and contextually grounded approaches is particularly pronounced, as prevention and intervention research rarely focuses on racialized girls.

Art therapy offers a culturally responsive approach for racialized youth who may struggle to verbalize complex and often silenced experiences^33^. By integrating symbolic, embodied, and creative methods, art therapy provides a flexible medium for meaning-making and emotional processing^34^. Evidence indicates that art therapy reduces symptoms of anxiety, depression, and trauma in marginalized adolescents and refugee populations^35–37^. Community-based art therapy programs have also demonstrated high feasibility and acceptability^38^.

Beyond symptom reduction, art therapy can foster resilience-related processes including self-efficacy, emotional regulation, and identity exploration^39,40^. In group formats, shared creative experiences strengthen belonging, validation, and solidarity^37,41^. For BIPoC girls, whose voices are often marginalized in institutional settings^42^, art-based practices can support narrative agency, the externalization of intergenerational trauma, and community connection^43,44^.

By fostering individual and group-based resources, art therapy aligns with the broader concept of resilience. Resilience, defined as the capacity to adapt and maintain well-being in the face of adversity^45^, is a key protective factor for BIPoC individuals navigating structural, institutional, and interpersonal racism^46,47^. Empirical evidence supports this relational understanding of resilience, with female-identifying BIPoC youth demonstrating strong stress management capacities during the COVID-19 pandemic^48^. Feminist-informed positive youth development models further conceptualize resilience not as an individual trait, but as a relational, contextual, and collective process^49^. Models on resilience among minority youth emphasize individual, family, and contextual resources, such as racial/ethnic identity, social support, and prosocial involvement, that can be strengthened through interventions^50^. Consistent with this, identity affirmation, self-care and self-awareness, and self-acceptance have been identified as key resilience-related factors for Black female adult populations^51–53^.

Despite promising international evidence for art-based and participatory interventions^41,54,55^, no studies in Germany have evaluated art-based interventions specifically tailored to BIPoC girls, and intersectionality-informed approaches remain rare. Given this absence, it remains unknown how such a program might be received by BIPoC girls and what short-term effects it may have on resilience-related resources. This gap positions the *Colors of Empowerment* intervention, developed through participatory adaptation, as the first of its kind in the German context. Grounded in resilience theory and feminist-informed positive youth development models, the intervention was co-designed with the target group to ensure cultural relevance and contextual fit.

The present proof-of-concept study examines:

- the needs and preferences of the target group towards a racism-sensitive, resource-oriented and art-based group intervention, identified through a focus group,
- the feasibility and acceptability of a self-reflective journal as an intervention component, piloted with female adolescents to examine usage patterns and perceived helpfulness and.
- the feasibility, acceptability, and preliminary psychosocial impact of the six-week art-based group intervention for BIPoC girls, including baseline assessments of resources and discrimination experiences to clarify participants’ needs.

To address these aims, a mixed-methods design was employed, combining quantitative pre/post and weekly measures with in-depth qualitative feedback. By centering BIPoC girls’ existing strengths, creativity, and sense of cohesion, the intervention positions these resources as key entry points for strengthening psychological resilience and informing culturally affirming group-based interventions in Germany.

## Methods

### Study design

**Fig. 1:**
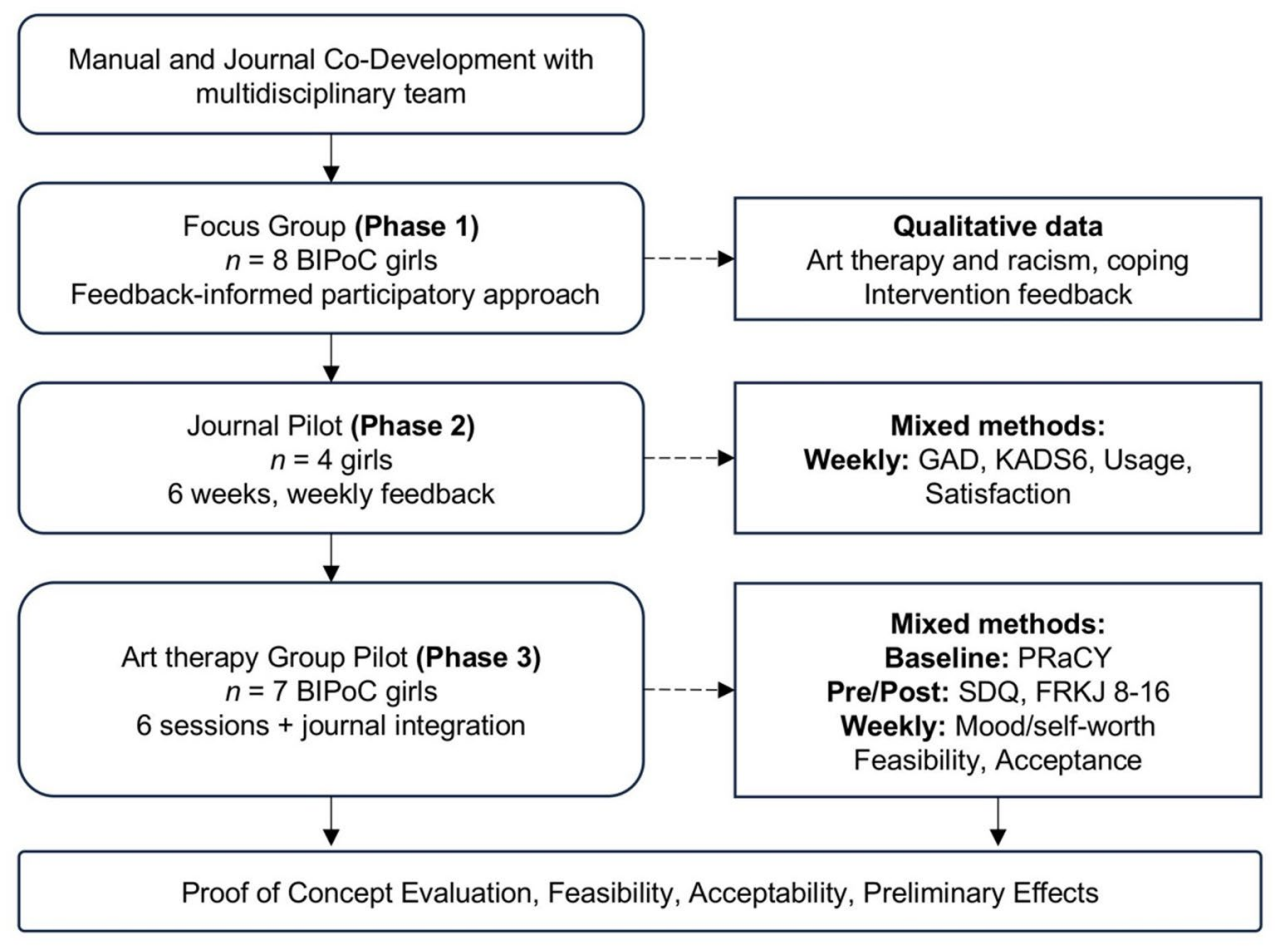
Overview of study phases, data collection, and evaluation points.

This mixed-methods proof-of-concept study developed and piloted a culturally responsive, intersectionality-informed and resource-oriented art therapy group intervention for BIPoC girls using a participatory approach. While grounded in participatory principles, this project does not claim to represent full participatory or co-produced research. Instead, participatory is understood here as meaningful youth involvement, focusing on integrating young people’s perspectives into the design and adaptation of the intervention. This framing aligns with conceptual work distinguishing different dimensions of youth participation that vary in purpose, positioning, power relations, and process^56,57^. The three sequential phases consisted of: (1) feedback-informed adaptation of a semi-structured art therapy manual and self-reflective journal through a focus group with BIPoC girls, (2) an independent six-week pilot of the self-reflective journal with female adolescents, and (3) a six-week group-based art therapy intervention for BIPoC girls using the manual and journal. The aim was to assess feasibility, acceptability, and preliminary effects of this resilience-oriented, identity-affirming approach. Recruitment took place through community networks, schools, and social media platforms. Written informed consent was obtained from all participants and, for those under 15 years of age, from their legal guardians as well. For an overview of the study phases and the measurements, see Fig. 1.

### Phase 1: Participatory adaption of the manual and reflective journal

#### Participants

Eight girls of Color (ages 14–16; *M* = 15.00, *SD* = 0.53), all of whom self-identified as BIPoC, participated in a 180-minute focus group. Participants were recruited through community networks and received €75 in compensation for their time and expertise.

#### Procedure

The focus group was conducted in a familiar community setting and co-facilitated by a White clinical psychologist and a BIPoC art therapist. The session followed a structured format including: (1) an introductory round and group agreement, (2) thematic discussions on experiences of racism, coping, and art-based support, (3) guided feedback on draft workshop content and journal materials, (4) small-group and plenary discussions to elicit concrete suggestions for adaptation, and (5) a closing reflection and summary.

Participants were invited to comment on the relevance of proposed themes, emotional pacing, tone and language, format, and specific exercises. They provided experiential perspectives and concrete recommendations regarding what felt helpful, inappropriate, or missing from the draft materials.

Following the focus group, the research team revised the structure, tone, and content of the manual and journal to reflect this feedback. While final decisions regarding feasibility and content integration remained with the interdisciplinary team, youth input directly informed key adaptations, including thematic emphasis, session flow, and the framing of sensitive topics. Detailed focus group procedures are provided in Supplement S1.

#### Data collection

The focus group was audio-recorded and transcribed verbatim. Field notes were taken to document group dynamics and non-verbal responses during discussions of sensitive topics.

#### Data analysis

Qualitative data were analyzed using Braun and Clarke’s reflexive thematic analysis^58,59^. Two researchers independently coded the transcripts inductively, focusing on participants’ experiences of racism, use of creative coping strategies, and preferences for supportive group-based interventions. Codes were discussed and refined collaboratively to develop themes that directly informed adaptation of the intervention materials.

### Phase 2: Reflective journal pilot testing

#### Participants

The six-week reflective journal pilot examined feasibility, usage patterns, and perceived helpfulness of the journal as a stand-alone intervention component. Four adolescent girls (ages 14–16) participated. Three participants reported being bilingual, with Russian as a second native language. Three indicated that they had “not at all or very rarely” experienced discrimination, while one reported experiencing discrimination “from time to time” in relation to her ethnic background. No information on self-identification regarding racial or ethnic identity was collected. Although the reflective journal was developed for use with BIPoC girls, Phase 2 relied on a convenience sample of female adolescents to assess basic feasibility and usability. This decision reflects the practical challenge of recruiting racialized youth for short-term pilot testing without established community partnerships. The Phase 2 data are therefore interpreted as preliminary indicators of feasibility rather than evidence of cultural fit for the target group. Each participant received a €20 voucher and individualized written feedback on their mental health data.

#### Procedure

**Fig. 2:**
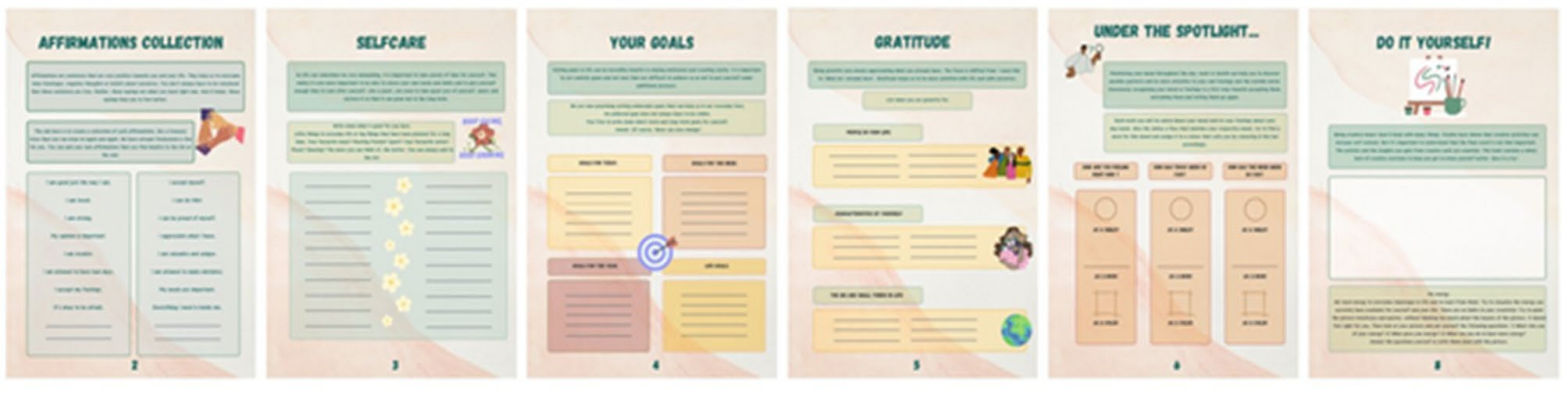
Extracts from the journal.

The pilot was conducted over six weeks. The journal’s six recurring sections (daily affirmations, self-care prompts, goal-setting, gratitude reflection, mood tracking, and art-based activities) aligned with the planned workshop themes and aimed to promote mindfulness, emotional regulation, and strengths-based reflection (see Fig. 2 for example extracts of the journal). Participants received either a printed (*n* = 3) or digital (*n* = 1) version of the reflective journal. One week before the start of the pilot, participants completed baseline questionnaires. They were then instructed to begin using the journal the following week. Participants were encouraged to engage with the journal once per week. Each week, they were asked to complete (a) the general tasks in the introductory section and (b) one set of week-specific tasks, which were assigned sequentially across the six-week period to ensure that all journal components were addressed. Each Thursday, participants received a short online questionnaire to be completed on Sundays. These weekly questionnaires functioned both as reminders to engage with the journal and as assessment tools for documenting usage patterns, satisfaction, and perceived emotional effects. After the sixth questionnaire, participants were no longer instructed to use the journal and completed post-assessments in the final study week.

No formal adherence criterion (e.g., minimum number of completed tasks) was defined, as the primary aim of this phase was to explore feasibility and acceptability rather than to evaluate effectiveness under standardized compliance conditions. Full procedural and measurement details are provided in Supplement S2.

#### Data collection

Data were collected using a combination of standardized self-report measures and study-specific questionnaires. One week prior to the start of the journal pilot, participants completed baseline assessments of anxiety and depressive symptoms using the Generalized Anxiety Disorder Scale-2 (GAD-2;^60^ and the Kutcher Adolescent Depression Scale-6 (KADS-6;^61^. During the six-week pilot phase, participants completed short weekly online questionnaires assessing frequency of journal use, number of days engaged, perceived helpfulness, and immediate emotional effects of journaling. These weekly questionnaires also served as structured reminders to support engagement with the journal tasks. Following the completion of the pilot, participants completed post-assessments that included the same standardized symptom measures as well as additional self-constructed items assessing usage patterns, perceived emotional impact, and satisfaction with the journal. Post-intervention feedback further comprised categorical and open-ended questions on reasons for use and non-use, as well as Likert-scale ratings of perceived changes in stress, self-acceptance, self-reflection, self-confidence, and mood.

#### Data analysis

Given the small sample size and feasibility focus, analyses were descriptive. Usage patterns, completion rates, and perceived helpfulness were summarized using means, standard deviations, and percentages. For each journal section, an accomplishment/helpfulness (A/H) ratio was calculated to examine the alignment between completion and perceived usefulness. Values close to 1.0 indicated strong alignment, values below 1.0 indicated higher completion than perceived helpfulness, and values above 1.0 indicated lower completion but high perceived usefulness. Pre - post changes in anxiety and depressive symptoms were summarized descriptively. No inferential statistics were conducted.

### Phase 3: Art-based group intervention pilot

#### Participants

Seven adolescent girls (ages 13–16; *M* = 14.14, *SD* = 1.07) participated in the six-week group-based art therapy intervention. All participants self-identified as BIPoC and were proficient in German. Written informed consent was obtained from all participants and, for those under 15 years of age, from their legal guardians. Participants received the reflective journal and a €20 expense allowance for completing the evaluation measures. See Table 1 for an overview of participant demographics and self-reported social identities across study phases.

**Table 1:** Participant racial/ethnic and gender identity across study phases.

	Phase 1 (*n* = 8) *n* (%)	Phase 2 (*n* = 4) *n* (%)	Phase 3 (*n* = 7) *n* (%)
Age M (SD)	15.00 (0.53)	15.25 (0.96)	14.14 (1.069)
Racial/ethnic identity		na	
Black/African descent	4 (50%)	na	2 (28.6%)
Mixed	1 (12.5%)	na	4 (57.1%)
Person of Color	3 (37.5%)	na	1 (14.3%)
White		na	
Gender identity			
Female	8 (100%)	4 (100%)	7 (100%)

M = mean; SD = standard deviation; Racial/ethnic categories are self-identified, were grouped for clarity and were reported through fixed-choice, open-text formats and self-assigned participation in the project.

#### Procedure

Participants attended six weekly group sessions of approximately three hours each. Sessions were co-facilitated by two clinical psychologists of Color and followed a non-directive art therapy approach. The intervention integrated guided art-making, group reflection, and optional use of the reflective journal for in-session and at-home practice. Each session focused on a thematic area related to identity, emotional processing, and resilience (see Table 2 for session themes and example exercises). The format emphasized psychological safety, narrative agency, and cultural sensitivity. Participants were invited, but not required, to share their artwork and reflections within the group.

Feasibility and acceptability were examined through session attendance, engagement with intervention components (including journal uptake), weekly satisfaction ratings, and structured post-intervention feedback.

**Table 2:** Overview of group sessions and example art-based exercises.

Session	Theme	Art-therapeutic exercise example
1	Self-perception and connection	Body scan and creation of a personal symbol on a group sheet, using placement, size, and color to reflect feelings and connections
2	Compassionate self-dialogue	Identify self-talk phrases, transform them into a feeling sketch, and develop personal affirmations
3	Building self-trust and self-efficacy	Meditation followed by a collage or drawing representing the multifaceted self
4	Emotional responses to racism	Short reflection on fear as a protective feeling, followed by creating an image of a door to explore what it represents (e.g., barriers, what’s behind it, feelings it evokes), and group reflection
5	Validating anger	Visualize emotions, release anger through clay throwing, and reshape into a desired form
6	Identifying personal and collective resources	Depict a moment of pride and draw it, share in the group, and brainstorm supportive resources

Each session started and ended with short rituals, e.g., reflection on the previous session and time in between, a brief self-check (“How am I feeling now?”), and identified takeaways. Exercises of all sessions included guided art-making, group reflection, and optional journal entries for at-home practice.

#### Data collection

Phase 3 employed a mixed-methods design to assess feasibility, acceptability, and preliminary psychosocial trends.

##### Feasibility and acceptability

Feasibility was operationalized via session attendance logs and self-reported engagement with the reflective journal. Acceptability was assessed via brief weekly paper–pencil ratings of satisfaction and perceived helpfulness, complemented by structured post-intervention feedback including Likert-style ratings and open-ended questions on overall experience, group atmosphere, perceived relevance, helpfulness of art-based activities, and suggestions for improvement. In addition, participants provided ratings of each journal section (e.g., enjoyment and helpfulness), enabling section-specific acceptability analyses.

##### Preliminary psychosocial outcomes (pre–post)

Pre- and post-assessments were conducted to explore potential changes in internal resources and mental health symptoms. Internal and external resources were measured with the German Questionnaire on Resources in Childhood and Adolescence (FRKJ 8–16; Lohaus and Nussbeck^62^, including the internal resource subscales optimism, self-efficacy, and self-worth, and the external resource subscales parental support, peer support, and school well-being. Items were rated on a 4-point scale (1 = never true, 4 = always true), and mean scores were calculated per subscale, with higher values indicating greater resources. Mental health symptoms were assessed using the Strengths and Difficulties Questionnaire (SDQ;^63^. SDQ subscale scores were categorized as normal, borderline, or abnormal using instrument guidelines and German normative cut-offs.

##### Contextual baseline measures (assessed once)

To contextualize participants’ starting conditions and needs, experiences of racism were assessed once using the Perceived Racism in Childhood and Youth Questionnaire (PRaCY;^5^. Participants also completed the short version of the Racial Trauma Scale (RTS;^64^. Given the adolescent sample, 8 of the 9 items were used, excluding the item “*Finding it difficult to cope without food/alcohol/drugs*.” RTS items were rated on a 4-point scale (1 = not at all, 4 = extremely), yielding total scores ranging from 8 to 32, with higher scores indicating greater racial trauma symptomatology. External resources (FRKJ subscales), experiences of racism (PRaCY), and racial trauma symptoms (RTS) were assessed only once to provide contextual information.

##### Weekly process measures

During the six-week intervention period, participants completed brief weekly assessments including current affect (PANAS;^65^, perceived everyday difficulties and perceived changes in these difficulties immediately after the session (adapted from the SDQ follow-up version;^66^, and self-created items assessing post-session self-satisfaction.

#### Data analysis

Given the proof-of-concept nature of the study and the small sample size, analyses were primarily descriptive.

Quantitative data from pre–post assessments and weekly measures were summarized using means, standard deviations, and score distributions. Within-subject effect sizes (Cohen’s *dz*;^67^ were calculated to describe the magnitude of pre–post changes in internal resources and mental health indicators. No inferential statistical testing was conducted.

To examine section-level acceptability of the journal in Phase 3, participants rated each section on fun and helpfulness, and a fun/helpfulness (F/H) ratio was calculated analogously to the accomplishment/helpfulness ratio used in Phase 2. Values below 1.0 indicated greater perceived helpfulness than enjoyment, while values above 1.0 indicated greater enjoyment than helpfulness.

Qualitative feedback from post-intervention questionnaires and session evaluations was analyzed using reflexive thematic analysis^58,59^. This qualitative material was used to contextualize quantitative trends and to explore participants’ experiences of feasibility, acceptability, and perceived impact of the intervention.

All quantitative analyses from Phase 1–3 were conducted in SPSS 29, and qualitative data were managed in MAXQDA (Version 24.5.1).

### Ethical considerations

The study was approved by the Ethics Committee of Freie Universität Berlin under the reference number 037/2023. All procedures followed were in accordance with the ethical standards of the 1964 Declaration of Helsinki and its later amendments. Additionally, the study was pre-registered on OSF to ensure transparency and adherence to the proposed methodology (10.17605/OSF.IO/XNMKZ*).* The reflective journal pilot (Phase 2) was conducted outside of this preregistration.

### Positionality

The authors acknowledge that their cultural identities, professional backgrounds, and positional power shape the research process, interpretation, and reporting of findings. Author 1 is a White cisgender woman, clinical psychologist, PhD candidate, and psychotherapist in training from Germany. She led the Colors of Empowerment project, secured funding, coordinated the art-based group therapies, and co-facilitated the focus group together with a BIPoC art therapist. Although she did not facilitate the therapeutic sessions herself, her dual role as researcher and project lead entailed positional power in relation to both the team and participants. To mitigate this, reflexive practices were employed, and therapeutic delivery was carried out by BIPoC art therapists. Author 2 is a PoC cisgender woman, clinical psychologist and psychotherapist in training from Germany. She served as one of the therapists delivering the therapeutic sessions within the Colors of Empowerment project. Her lived experience as a PoC provided insider competence in working with the target population. However, her dual role as therapeutic facilitator and researcher created potential role conflict that could have influenced data collection. This was addressed through reflection and collaborative interpretation of findings. Author 3 is a White cisgender woman from Germany, a licensed child and youth psychotherapist (CBT) and the PhD supervisor of Author 1. While not directly involved in project coordination or group facilitation, her supervisory role entails academic authority and positional power in shaping the research framing and interpretation.

## Results

### Phase 1: Focus group findings

Using reflexive thematic analysis, four main themes and related subthemes were identified, describing how participants experience and respond to racialized stress, their encounters with art therapy, and their preferences for supportive, creative interventions (see Table 3).

### Theme 1: Processing racism through art

Participants described creative practices as safe outlets for expressing and reflecting on emotions (*Expressing Feelings*), especially when verbalizing was difficult:

Sometimes you just don’t feel like talking to others and want to be alone. When there’s a lot of anger inside, it might be better to take a quiet approach. You could listen to music or draw by yourself.

Art was also seen as a way to make racialized experiences visible (*Visibility*) and raise awareness, with ideas such as public art walls. Finally, participants valued having multiple formats (*Using Different Forms*) like music, theater, and podcasts to accommodate personal preferences.

### Theme 2: Art therapy experiences

Participants’ familiarity with art therapy varied (*Familiarity*), with some using art informally for self-regulation and others encountering the concept for the first time. Personal interest also differed but seemed important for engagement (*Personal Interest*), while past school-based experiences sometimes felt unsafe (*Contextual Factors*). Therefore, small, familiar groups were preferred:

I think something like this shouldn’t be done in a big group. Smaller groups are much more effective. It helps if you already know the others a little bit.

### Theme 3: Experiences of racism and coping

Accounts included both subtle and overt racism (*Subtle and Overt Racism*) and the inner debate over whether to respond (*Emotional Conflict*):

When something like that happens on the street, I always ask myself: ‘Should I say something or just walk away?’ Because you won’t see them again anyway. But sometimes, after I leave, I think: ‘I should’ve said something.’ Because maybe if I had, they wouldn’t have done it again. Maybe it would’ve made them think. But often I just get so angry and then I don’t say anything […].

Coping strategies ranged from confrontation to emotional detachment (*Coping Mechanisms*). Support from trusted relationships (*Seeking Support)* was central:

I like to talk to people I trust - friends or family. Just talking helps. And maybe you don’t have to talk about it right away, only when you’re ready.

### Theme 4: Workshop preferences

Participants recommended shared experiences among participants and culturally sensitive facilitators (*Group Composition and Facilitators*), gender-specific safety (*Gender-Specific Safety*), gradual introduction of sensitive topics (*Content Relevance and Emotional Pacing*), and holding sessions in voluntary spaces outside of potentially discriminatory environments, e.g. schools (*Structural Preferences)*. On group composition and facilitators, one participant emphasized:

What matters is who offers the workshop […] that the facilitators are friendly. And that the participants are in the same age group and ideally, also have a migration background.

**Table 3:** Overview of themes, subthemes, and illustrative quotes from the focus group with BIPoC adolescent girls.

Main theme	Subtheme	Short description	Illustrative quotes
Processing Racism through Art	Expressing Feelings	Art used for emotional regulation and reflection	*“…then I drew something… the way I felt…”*
	Visibility	Art makes experiences of racism visible to others	*“Maybe if famous people talked about it…”*
	Using Different Forms	Podcasts, theater, music suggested as alternatives	*“You could even do a podcast…”*
Art Therapy Experiences	Familiarity	Varied awareness of art therapy; some first contact	*“I’ve never heard the term art therapy…”*
	Personal Interest	Engagement linked to enjoyment of art	*“Art isn’t really my passion…”*
	Psychological Safety	Small groups and known peers foster comfort	*“It helps if you already know the others…”*
Experiences of Racism and Coping	Subtle/Overt Racism	Racism experienced in public spaces, school, family	*“She just stared at me the whole time…”*
	Emotional Conflict	Struggles with responding or staying silent	*“Should I say something or just walk away?”*
	Coping Strategies	Responses range from confrontation to detachment	*“Over time you just don’t care anymore.”*
	Support from Others	Talking with family/friends is key coping strategy	*“Just talking helps…”*
Workshop Preferences	Group/Facilitators	Need for shared identity among group and facilitators	*“Ideally… have a migration background.”*
	Gender-Specific Safety	Female-only groups seen as important	*“Make sure that a woman leads the workshop…”*
	Emotional Pacing	Difficult topics should be introduced gradually	*“Start with easy things*,* then go deeper.”*
	Structure	Prefer non-school spaces, breaks, and shorter sessions	*“Not at school… otherwise it’s too forced.”*

Main themes and subthemes were identified through reflexive thematic analysis of a 180-minute focus group with eight BIPoC girls (14–16). Subthemes were developed inductively and illustrated with quotes, lightly edited for clarity while preserving meaning. All quotes were translated from German to English by the authors.

### Phase 2: Journal Pilot

As described in the Methods, the journal pilot was conducted with a convenience sample of female adolescents rather than the intended target group of BIPoC girls. The findings from this phase therefore primarily inform feasibility, usability, and engagement patterns, while conclusions regarding cultural relevance rely mainly on data from the subsequent group pilot in Phase 3. On average, participants engaged with the journal for 49.9 min (*SD* = 72.96) across 2.13 days (*SD* = 1.29) per week. The very high variability in engagement time indicates substantial heterogeneity in how participants used the journal, ranging from minimal engagement to more intensive use. Across all weekly assessments (*n* = 24 responses), 66.6% of responses (*n* = 16) rated the journal as helpful, 25% (*n* = 6) as sometimes helpful, and 8.3% (*n* = 2) as not helpful. Reported motivations for usage included emotional relief, creative expression, and taking a break from daily stress (“*To get thoughts out of my head and onto paper”*), while fatigue and lack of time were the most common barriers (21.9% of responses, *n* = 5). More than half of weekly responses (58.3%, *n* = 14) indicated the journal helped participants perceive themselves differently, typically in a more positive way (e.g., *“I became more aware of my achievements”*). Immediate post-journaling effects as reported in open-ended responses, included feeling relaxed, liberated, or cared for, although general mood changes on journaling days were mixed and sometimes short-lived. Changes in mental health symptoms and internal resources after the 6 week journal pilot are descriptively presented in Supplement S3 and Supplementary Figure S1.

Regarding the specific journal sections, completion was highest for affirmations (96%, *n* = 23), gratefulness (100%, *n* = 24), and self-care (100%, *n* = 24), which also received the highest helpfulness ratings (75%, *n* = 18; 66.6%, *n* = 16; and 62.5%, *n* = 15 respectively). Exercises requiring extra materials were completed less often but still valued positively by some participants. Across sections, higher accomplishment/helpfulness (A/H) ratios reflected greater feasibility and perceived helpfulness (see Supplementary Table S2). Participants highlighted the reflective and emotional impact of certain tasks, e.g., *“They showed me what I can be grateful for and helped me appreciate things more.”*

### Phase 3: Workshop Intervention Pilot

#### Feasibility

The group-based setting and session structure were implemented as planned. Attendance was high, with all seven participants attending at least half of the sessions and *n* = 5 (71%) attending four or more of the six sessions. Participants suggested two minor adjustments to improve delivery, namely allowing more time for creative activities and longer introductory rounds to foster connection, while also noting a logistical aspect concerning snack variety.

Journal uptake was limited. Four of the seven participants (57%) reported not using the reflective journal at all, two reported rare use (1–2 times total), and one participant reported occasional use.

#### Acceptability

Weekly evaluations indicated high overall satisfaction. Across all sessions, 26 of 28 session ratings (92.9%) corresponded to school grades of 1 or 2 (1 = excellent). Regarding immediate effects, participants reported feeling better after the session in 21 of 28 (75%) session evaluations, while in 7 evaluations (25%) they indicated feeling the same as before. Regarding content relevance, five participants (71%) felt that the workshop addressed their personal needs “well” or “very well,” while two participants (29%) indicated that it addressed their needs “rather not.” Post-intervention, the group atmosphere was described positively overall: five rated it as “very pleasant,” or “pleasant,” one described it as “okay” and one participant provided a neutral rating.

Across the 28 session-based qualitative feedback responses, creative art exercises were most frequently highlighted as positive aspects (*n* = 16; 57%), including activities such as painting, throwing clay, and collage-making. Interpersonal connection and shared dialogue were also valued (*n* = 7; 25%), with participants noting “the exchange of our experiences,” “getting to know new people,” and “the conversations.” Snacks and the relaxed atmosphere were mentioned in 5 of 28 responses (18%).

Several participants described positive changes over time, e.g., “It was a bit stressful at first, but that went away,” or learning that they were “not alone with the everyday problems as a PoC person.” At the end of the intervention, *n* = 3 participants stated they would recommend the workshop, while *n* = 4 were uncertain.

The reflective journal was evaluated less positively than the group sessions. Of the seven participants, two rated the journal as helpful, two as okay, one as not so helpful, and two as not helpful at all. The mean helpfulness rating was 3.43 (1 = *very helpful*, 5 = *not helpful at all*). Ratings (1–10) across the specific journal sections indicated moderate enjoyment (*M* = 4.35), usefulness (*M* = 4.40), and importance (*M* = 5.00). Ratings of individual journal sections showed that Self-Care, Goal Setting, and Art Therapy were rated highest for enjoyment, while Monitoring, Self-Care, Goal Setting, and Blank Lines scored highest for helpfulness. Ratios indicated that Monitoring was rated as more helpful than enjoyable (0.59), while Gratefulness was rated as more enjoyable than helpful (1.43). Self-Care, Affirmation, and Goal Setting showed balanced ratios (~ 1.0). Full descriptive ratings and mismatch indices for all journal sections are presented in Supplementary Table S3.

### Preliminary effects on resources and mental health

#### Baseline values: Internal resources, mental health, and discrimination exposure

At baseline, participants reported moderate levels of racial trauma (*M* = 17.00, *SD* = 3.11; range = 11–21) alongside limited internal resources. On a 1–4 scale, mean scores for optimism (*M* = 2.24, *SD* = 0.52), self-efficacy (*M* = 2.07, *SD* = 0.53), and self-worth (*M* = 2.09, *SD* = 0.50) indicated that these strengths were experienced only sometimes. External resources were rated slightly higher, with mean scores of 2.71 (*SD* = 0.66) for parental support, 2.88 (*SD* = 0.56) for peer support, and 2.64 (*SD* = 0.69) for school well-being. Regarding mental health, emotional problems on the SDQ were slightly elevated (*M* = 5.43, *SD* = 2.94), whereas conduct problems fell within the average range (*M* = 2.43, *SD* = 0.98).

All participants (*n* = 7; 100%) reported experiencing at least one discriminatory event on the PRaCY. On average, they endorsed nearly half of the listed situations (*M* = 4.71 out of 10, *SD* = 1.38). The most frequent were being called insulting names (100%), unfair treatment by teachers (*n* = 4; 57.1%), and witnessing racism against family members (*n* = 6; 85.7%). These experiences were attributed primarily to race, skin color, ethnicity, accent, or gender. Emotional responses included anger, sadness, frustration, and powerlessness. Coping strategies ranged from ignoring, accepting to actively addressing or discussing the incident (see Table S4 in the Supplements).

#### Pre-post descriptive changes

No inferential statistics were conducted due to the small sample. Descriptively, we observed small to medium effect sizes ranging from 0.15 (self-worth) to 0.65 (self-efficacy) with descriptive increases in self-efficacy, self-worth, and optimism. Emotional and conduct problems increased slightly (see Table 4).

**Table 4:** Descriptive means and standard deviations of psychological variables at T0 and T1.

	Baseline (T0) M (SD)	Post (T1) M (SD)	Effect size Cohen’s dz
Optimism	2.24 (0.52)	2.33 (0.48)	0.18
Self-efficacy	2.07 (0.53)	2.43 (0.57)	0.65
Self-worth	2.09 (0.50)	2.17 (0.57)	0.15
Emotional problems	5.43 (2.94)	6.43 (1.62)	0.42
Conduct problems	2.43 (0.98)	3.00 (1.83)	0.39

M = mean; SD = standard deviations. Higher values on SDQ subscales reflect greater problem severity; higher values on resource scales indicate stronger perceived resources.

### Weekly affect and workshop experience

PANAS data showed no clear trend across six sessions. Positive affect ranged *M* = 2.62–3.37, while negative affect remained stable (*M* = 2.15–2.78; see Supplementary Figure S2 for a visual representation).

#### Everyday difficulties and coping support

Across the 28 session-level responses, most indicated the presence of everyday difficulties: 14 responses reported mild everyday difficulties, 7 responses reported significant difficulties, while 6 responses indicated none. Despite these ongoing challenges, participants frequently perceived the workshop as supportive for coping. Across sessions, 19 of 28 responses rated the workshop as rather or very helpful for dealing with problems, while 9 responses indicated little or no helpfulness.

When asked about short-term change in difficulties after sessions (22 valid responses), 13 responses indicated that problems stayed about the same, 5 responses reported that difficulties felt a little better, and 2 responses that they felt much better; 2 responses indicated slightly worse difficulties.

Reports of feeling better immediately after sessions increased over time, from 1 of 6 responses in Session 2 to 2 of 6 responses in Session 6, suggesting a modest upward trend in perceived short-term benefit.

Self-satisfaction was assessed at the end of each session using a 0-100 visual analog scale. Across 27 valid paired responses, participants rated their current self-satisfaction (“today”) on average 10.4 points higher than their past-week satisfaction (*SD* = 16.3; range − 20 to + 50).

## Discussion

To our knowledge, this is the first participatory art-based group intervention, developed and piloted specifically for BIPoC girls in Germany. Addressing the urgent need for intersectional and community-based approaches to promote resilience in marginalized youth^68^, this proof-of-concept study provides initial insights into feasibility, contextual relevance and perceived value. While conclusions regarding effectiveness must remain cautious, the findings suggest that creative, culturally responsive group formats may offer meaningful entry points for supporting psychological well-being and intrapersonal resources among BIPoC girls.

### Phase 1: Needs, resources, and contextual relevance

Findings from Phase 1 underscored the importance of connectedness, community, and emotional safety in coping with racialized stress. Participants emphasized the need for spaces where their experiences were seen, validated, and shared — conditions that enhance social resilience and buffer the psychological impact of racism^69,70^. This emphasis is consistent with resilience theory, which conceptualizes resilience as a dynamic process emerging from interactions between individual strengths and supportive environments^45^. It also reflects research on racial stress, which underscores the importance of collective care, anti-silencing, and belonging^71,72^.

Participants further described art as a meaningful medium to make experiences of racism visible and emotionally processable. These insights are in line with research showing that creative expression can foster narrative agency and identity development among marginalized youth^41,54,73^. Importantly, participants’ motivation and interest in creative activities emerged as a prerequisite for engagement in art-based interventions. This finding aligns with an ethnographic study showing that vulnerable youth were more receptive to art-making when they genuinely enjoyed creative processes^33^. Beyond the medium itself, participants highlighted the importance of contextual conditions such as cultural resonance, emotional safety, and non-judgmental engagement. Together, these elements underscore the relevance of a strength-based, culturally grounded group format and reflect core principles of resilience-informed art therapy, including identity development, resource activation, and interpersonal trust and safety^74,75^.

### Phase 2: Reflective journal as a supportive component

In Phase 2, the reflective journal demonstrated high feasibility and was perceived as emotionally engaging and accessible. At the same time, the marked variability in engagement observed in Phase 2 suggests that reflective journaling may resonate differently across adolescents, underscoring the importance of offering it as a flexible, optional component rather than a standardized requirement within arts-based interventions. Participants frequently described it useful for short, structured practices such as gratitude, affirmations, and goal-setting. These observations are consistent with positive psychology literature linking such practices to improvements in emotion regulation, mood, self-efficacy, and resilience in adolescents^76–80^.

Mindfulness-oriented sections of the journal appeared to support calmness, awareness, and reduced screen time. These subtle changes can be understood as everyday forms of resilience-building^81^, particularly in the context of coping with racism-related stress^82,83^. Congruent with Phase 3 findings, participants rated sections such as “Self-Care,” “Affirmation,” and “Goal Setting” as both helpful and enjoyable, This convergence suggest that journaling may function as a that journaling low-threshold, complementary component when embedded within structured group interventions.

### Phase 3: Feasibility, acceptability, and baseline context

Baseline data from Phase 3 highlighted the relevance of culturally responsive interventions. All participants reported prior experiences of racial discrimination, both direct and vicarious, including insults, teacher bias, and institutional racism. Moderate levels of racial trauma symptoms were observed, along with slightly elevated emotional problems and reduced self-worth, self-efficacy, and optimism. These patterns are consistent with research linking structural and interpersonal racism to diminished internal protective factors^84^.

Regarding feasibility and acceptability, participant feedback highlighted the value of shared experiences, collective reflection, and embodied art-making. Overall evaluations were positive, with most ratings indicating high satisfaction and the majority of participants reporting feeling better after sessions. At the same time, fewer indicated that they would recommend the workshop to peers. This discrepancy may reflect group-specific dynamics, stigma related to mental health interventions^85^, or ambivalence about sharing racialized experiences of within newly formed groups. Similar tensions between appreciation and ambivalence have been noted in other community-based art therapy programs^38^.

Participants also expressed a preference for greater flexibility and multimodality, particularly through the inclusion of music alongside visual arts. This aligns with prior research highlighting the role of music in fostering racial identity, empowerment, and emotional processing among youth of Color^86^. These preferences point to potential directions for refining future iterations of the intervention.

### Psychosocial trends in context

Preliminary descriptive outcome data suggested modest indications of improvements in self-efficacy and self-worth, alongside a slight increase in optimism. At the same time, emotional and conduct problems appeared to increase over the intervention period. Given the small sample size and descriptive nature of the analyses, these trends should be interpreted cautiously.

One plausible interpretation is that the intervention may have fostered heightened emotional awareness rather than symptom exacerbation. Emotions such as anger often marginalized or pathologized in BIPoC girls^24^—were explicitly addressed in the workshop and emerged across several exercises and discussions. This approach aligns with earlier art therapy work emphasizing the therapeutic relevance of anger and the need for structured, validating expression^70,87^.

Group art therapy processes may evoke complex and sometimes contradictory experiences, where participants feel both supported and unsettled, or empowered and vulnerable^88^. In line with this, weekly affect ratings fluctuated across sessions rather than following a linear trend, while qualitative responses provided contextual insight into how participants made sense of these fluctuations. Participants highlighted the perceived helpfulness of creative and group-based components, as well as short-term increases in self-satisfaction after sessions. Together, these findings illustrate how mixed-methods design can capture nuanced and non-linear change processes that may be overlooked by standard pre-post assessments^89^.

### Implications

#### Implications for mental health practice

Mental health practitioners working with racialized adolescents may consider incorporating structured, arts-based methods to support expression and meaning-making when racism-related experiences are difficult to verbalize. In practice, this can include brief creative warm-ups, externalization exercises (e.g., “mapping” stressors and resources), and identity-affirming art prompts (e.g., exploring personal strengths, cultural symbols, or future selves) that allow young people to control what is disclosed and the point at which they feel ready to share. Given participants’ emphasis on emotional safety and cultural resonance, clinicians may also treat the therapeutic context itself as central to the intervention by explicitly establishing group agreements, naming confidentiality boundaries, and adopting a non-judgmental, validating stance toward emotions such as anger that are often pathologized in BIPoC girls.

These findings further suggest the value of pairing creative work with brief, easy-to-implement self-regulation practices (e.g., short grounding or affirmation routines) to support between-session coping. Clinicians may also consider integrating brief, routine check-ins (including process-focused ratings) to acknowledge the non-linear nature of change and to respond flexibly to fluctuations in affect. Finally, participants’ ambivalence about recommending the workshop to peers may reflect concerns about how participation could be interpreted by others, particularly fears that the program might be seen as indicating vulnerability or “having problems.” Such stigma-related concerns can make young people hesitant to endorse the intervention publicly. This, in turn, highlights the importance of clearly communicating the aims of participation—namely empowerment, connection, and coping—so that the program is framed as a resource-oriented opportunity rather than as deficit- or problem-focused support. In addition, offering multiple pathways for engagement (e.g., speaking, writing, creating, or observing) may help accommodate different comfort levels within group settings.

#### Implications for preventive community- and school-based interventions

Given that experiences of racism frequently occur in everyday settings such as schools and peer environments, community- and school-based programs may represent particularly relevant contexts for implementing art-based group interventions. Low-threshold, group-based formats that combine creative expression with shared reflection may help reduce access barriers and normalize conversations about racism and mental health within these settings.

Beyond content, the findings point to the importance of how such interventions are embedded. Implementing arts-based programs in familiar environments (e.g., schools, youth centers, community organizations) may increase reach, while co-facilitation by trusted staff or community partners may help foster emotional safety and engagement. Community- and school-based stakeholders may therefore consider arts-based group formats not only as therapeutic add-ons, but as preventive and capacity-building spaces that complement existing psychosocial support structures for racialized youth.

#### Implications for future research

As a proof-of-concept study with a small sample size, the present findings highlight several directions for future research. Larger studies with longer follow-up periods are needed to examine the sustainability of psychosocial changes. Process-oriented and repeated assessments may be particularly useful for capturing non-linear change trajectories in arts-based group interventions.

Building on the participatory and arts-based orientation of the present study, future research may benefit from exploring additional multimodal pathways for capturing lived experiences and change processes over time. Approaches that allow participants to engage through visual, symbolic, and narrative modes may be particularly well suited for examining racism-related stress and resilience, which are not always readily accessible through verbal accounts alone. Online Photovoice (OPV), a participatory method that uses participant-generated images to support reflection and dialogue, could be used to complement in-session creative work by documenting experiences that emerge between sessions and in everyday contexts (Tanhan & Strack, 2020).

Future research would further benefit from qualitative and participatory methodologies that center the voices and lived experiences of racialized youth. Community-Based Participatory Research (CBPR) offers a framework for co-developing research questions, methods, and dissemination strategies in collaboration with participants and community partners, thereby enhancing cultural relevance and equity in research on racism and mental health^90,91^. Together, these directions point to the need for future research that combines adequately powered designs with participatory, multimodal methods to more fully capture how arts-based interventions shape resilience processes over time in the context of racism-related stress.

## Limitations

Although youth perspectives meaningfully informed the adaptation of materials, participation in Phase 1 was limited to a single consultation round and did not involve shared decision-making across all stages of intervention development. Future research may benefit from more sustained and multi-stage participatory approaches. Moreover, the reflective journal was piloted in Phase 2 with adolescents who did not primarily identify as BIPoC, limiting conclusions about cultural relevance for the intended target group. This phase focused on feasibility and usability testing under low-risk conditions, while insights into relevance for BIPoC girls derive mainly from the group-based pilot in Phase 3. The reliance on a convenience sample reflects the difficulty of reaching racialized youth for short-term feasibility studies without sustained community partnerships. The small sample size is the central limitation and prevents any generalization of findings. Despite broad recruitment via schools, NGOs, and social media, engagement remained challenging. Barriers may include mistrust toward institutional interventions among racialized communities and competing demands on adolescents’ time; however, we did not systematically assess reasons for non-participation nor non-recommendation of the intervention. The absence of a control group further restricts interpretability of observed psychosocial changes. Nevertheless, given that this proof-of-concept study primarily sought to evaluate feasibility and acceptability, the findings remain informative for guiding future intervention development. Future implementations should prioritize trust-building strategies, such as sustained community partnerships, and outreach through peers and youth advocates embedded in participants’ social networks.

## Conclusion and future directions

This pilot study provides initial evidence for the feasibility and relevance of a resilience-oriented art-based group intervention for BIPoC girls in Germany. It highlights the value of culturally responsive, emotionally safe, and creative approaches to supporting youth exposed to racialized stress, with promising indications for fostering self-reflection, emotional awareness, and short-term gains in self-efficacy and self-worth. The reflective journal, though used inconsistently, was perceived as a helpful, low-threshold tool for emotional regulation and may be more effective when integrated into in-session practice.

A central strength of the program was co-facilitation by BIPoC art therapists, which participants emphasized as key for safety, cultural resonance, and trust. These insights directly address the lack of intersectionality-informed, culturally attuned interventions for BIPoC girls in Germany. The present findings suggest that translation into practice is both feasible and timely.

## Supplementary Information

Below is the link to the electronic supplementary material.

Supplementary Material 1

## Data Availability

Due to the small sample size and the sensitivity of participant data, the datasets generated and analyzed during the current study are not publicly available. Further details can be requested from the corresponding author.
