# Supplementary material for: Participatory development and proof-of-concept of an intersectionality-informed art-based group intervention for BIPoC girls
Source: Sci Rep. 2026 Feb 17;16:6710. doi: 10.1038/s41598-026-40521-7 (PMC12913595; doi:10.1038/s41598-026-40521-7)

Supplement S1

The Phase 1 participatory focus group was designed to gather lived-experience input from BIPoC adolescent girls to inform the co-development of the workshop manual and reflective journal. Eight adolescent girls (14-16 years) who self-identified as Black, Indigenous or People of Colour (BIPoC) were recruited via youth organizations and school networks. All participants provided written informed consent. The session was co-facilitated by an art therapist and a clinical psychologist. Both facilitators identified as women, with at least one identifying as BIPoC. The focus group took place in person in a community space familiar to participants, with arrangements to ensure privacy and comfort. It lasted 180 minutes, including breaks. Light snacks and drinks were provided.

Procedure

The session combined semi-structured discussion with participatory creative methods to encourage open sharing and equal participation. Discussion was organized around four main topic areas:

1. Processing racism through art
2. Art therapy experiences
3. Experiences of racism and coping strategies
4. Workshop preferences

Supplement S2

The six-week reflective journal pilot involved four adolescent girls (M = 15.25 years, *SD* = 0.83; range 14-16).

Journal structure

The journal consisted of six recurring components aligned with the planned workshop themes:

1. Daily affirmations
2. Self-care prompts
3. Goal-setting
4. Gratitude reflection
5. Mood tracking (daily, weekly)
6. Art-based activities (e.g., free drawing, creative prompts)

These were designed to encourage mindfulness, emotional regulation, and strengths-based reflection.

Data collection and Measurements

Participants completed weekly questionnaires, containing journal usage (days per week, minutes per day), reasons for use/non-use, satisfaction ratings, and perceived emotional effects (immediate and general mood impact). Furthermore, we assessed pre-post questionnaires: demographic data (pre only) and standardized measures of anxiety (GAD-2; [56]) and depression symptoms (KADS-6; [57]). GAD-2 and KADS-6 items were rated on a 4-point scale from 1 = not at all to 4 = almost every day. Perceived overall helpfulness of the journal was rated on a 3-point scale (yes,

sometimes, no). Section-specific accomplishment and helpfulness were assessed via binary yes/no items, with optional written explanations. Additional effectiveness-related items (e.g., "*The journal has helped me accept myself more*") were rated on a 5-point scale (1 = strongly disagree to 5 = strongly agree).

Analysis

Quantitative data were analyzed descriptively. Qualitative responses were coded inductively to identify the feasibility, perceived usefulness, and potential effects of the self-reflective journal.

Supplement S3

Mental Health and Internal Resources

Over the pilot period, descriptive trends indicated slight decreases in anxiety and depression and increases in perceived well-being, self-reflection, and self-acceptance during the six weeks (see Figure S1).

Figure S1. Changes in Mental Health and Internal Resources During Journal Pilot

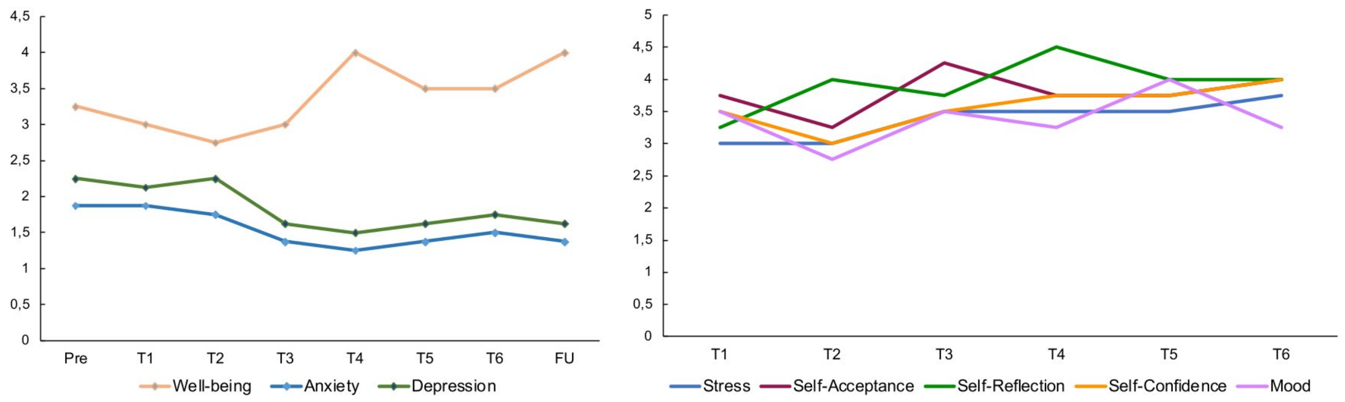

Supplementary Table S2.

Completion Rates, Perceived Helpfulness, and Accomplishment/Helpfulness Ratios for Journal Sections

Section	Accomplished[a] (A)<br>*n* (%)	Helpful[a] (H)<br>*n* (%)	A/H Ratio
Monitoring 1: feeling[b]	24 (100%)	6 (25%)	.25
Monitoring 2: day[c]	24 (100%)	10 (41.7%)	.42
Monitoring 3: week[d]	24 (100%)	8 (33.3%)	.33
Gratefulness	24 (100%)	16 (66.6%)	.67
Affirmation	23 (96%)	18 (75%)	.78
Self-Care	24 (100%)	15 (62.5%)	.62
Goal Setting	24 (100%)	16 (41.7%)	.67
Art therapy I	12 (50%)	5 (20.8%)	.42
Art therapy II	7 (29%)	4 (16.7%)	.57
Blank lines	19 (79%)	10 (41.7%)	.53

Notes. a = Number of participants who answered "yes" (i.e., completed the exercise or found it helpful). A/H Ratio = Ratio of completed exercises to those rated as helpful. Monitoring: b = "How are you feeling today?", c = "How was your day so far?", d = "How was your week so far?"

Supplementary Table S3.

Descriptive Ratings and Mismatch Indices for Journal Sections (N = 7)

Journal Section	Fun<br>(*M*, *SD*)	Helpfulness<br>(*M*, *SD*)	Ratio F/H*	Summary Mismatch
Monitoring	3.75 (4.11)	6.33 (5.51)	0.59	High
Gratefulness	4.75 (4.11)	3.33 (4.93)	1.43	High
Affirmation	4.75 (3.78)	4.50 (6.36)	1.05	Low
Self-Care	5.25 (4.11)	5.00 (5.00)	1.05	Low
Goal Setting	5.25 (4.11)	5.00 (4.34)	1.05	Low
Art therapy I	5.00 (3.32)	4.33 (4.51)	1.15	Moderate
Art therapy II	4.00 (3.74)	4.67 (4.51)	.86	Moderate
Blank lines	4.00 (3.74)	5.00 (4.58)	0.8	Moderate
Workshop Information	3.75 (3.86)	4.00 (4.00)	0.94	Low
Workshop Take Home	3.25 (3.403)	4.00 (4.00)	.81	Moderate

Note. Ratings ranged from 0 (not at all) to 10 (very much). * The ratio F/H reflects the relationship between reported fun and helpfulness for each journal section, with values < 1 indicating higher helpfulness than fun, and values > 1 indicating higher fun than helpfulness.

Supplementary Table S4.

Descriptive Overview of Reported Racism Experiences at Baseline

Item	% Experienced Event	Attributed to Discrimination (*n*)	Frequency of Occurrence (*n*)	Emotions Reported (*n*)	Coping Strategies (*n*)
Watched closely or followed in a store	42.9% (*n* = 3)	Race (2), Ethnicity (1), Skin color (1), Gender (1)	Once, once/year, once/month	Angry (1), Annoyed (2), Hurt (3), Frustrated (3), Sad (2), Depressed (1), Hopeless (1), Powerless (3)	Ignored (3), Addressed (1), Kept for self (1), Lost interest (1), Tried to change (1), Tried to forget (1)
Accused of something at school	42.9% (*n* = 3)	Ethnicity (2), Skin color (2), Gender (2)	Once, twice, once/year	Angry (2), Annoyed (2), Hurt (2), Frustrated (1), Sad (1), Hopeless (1), Powerless (1), Nothing (1)	Ignored (1), Accepted (2), Lost interest (1), Tried to forget (1), Worked hard (1)
Treated unfairly by a teacher	57.1% (*n* = 4)	Race (1), Ethnicity (1), Skin color (1), Gender (1)	Once/year (1), Once/month (2), Weekly (1)	Angry (2), Annoyed (2), Hurt (3), Frustrated (2), Sad (2), Depressed (1), Hopeless (1), Powerless (1), Exposed (1)	Ignored (1), Accepted (1), Addressed (3), Kept for self (1), Lost interest (1), Tried to change (1), Tried to forget (1), Worked hard (1)
Someone was afraid of you	14.3% (*n* = 1)	Skin color (1), Language (1)	Once/month	Hurt (1), Frustrated (1), Sad (1)	Ignored (1), Accepted (1), Tried to forget (1)
Called an insulting name	100% (*n* = 7)	Race (3), Ethnicity (2), Skin color (4), Accent (1), Gender (3)	Once (1), Twice (1), Once/month (4), Weekly (1)	Angry (5), Annoyed (4), Hurt (3), Frustrated (2), Sad (3), Depressed (2), Hopeless (3), Powerless (3)	Ignored (2), Accepted (4), Addressed (4), Kept for self (2), Lost interest (1), Tried to change (2), Tried to forget (2), Discussed (1)
Heard insulting remarks about race/ethnicity	57.1% (*n* = 4)	Race (1), Ethnicity (3), Skin color (3), Gender (3)	Once/year (1), Once/month (2), Weekly (1)	Angry (1), Annoyed (1), Hurt (2), Frustrated (1), Sad (1), Hopeless (1), Powerless (1), Strengthened (1), Bored (1)	Ignored (1), Accepted (1), Addressed (2), Tried to forget (1)
Someone was rude to you	71.4% (*n* = 5)	Ethnicity (1), Skin color (2), Gender (2)	Once/year (1), Once/month (4)	Angry (2), Annoyed (1), Frustrated (1), Sad (2), Depressed (1), Hopeless (1), Powerless (1)	Ignored (3), Accepted (2), Addressed (1), Kept for self (1), Tried to forget (1)
Family treated unfairly due to background	85.7% (*n* = 6)	Race (2), Ethnicity (3), Skin color (4), Language (2), Accent (2)	Once (2), Twice (2), Once/month (2)	Angry (5), Annoyed (3), Hurt (3), Frustrated (2), Sad (2), Hopeless (1), Powerless (1)	Ignored (2), Accepted (3), Addressed (2), Lost interest (1), Tried to forget (1)

Note. The table summarizes participants' reported experiences of discrimination, including type and context of the event, perceived attribution (e.g., race, ethnicity, gender), emotional responses, and coping strategies. Percentages reflect the proportion of participants endorsing each experience (*N* = 7). Participants could report more than one attribution, emotion, and coping strategy per event.

Figure S2. Mean Positive (PA) and Negative Affect (NA) Ratings Across Sessions

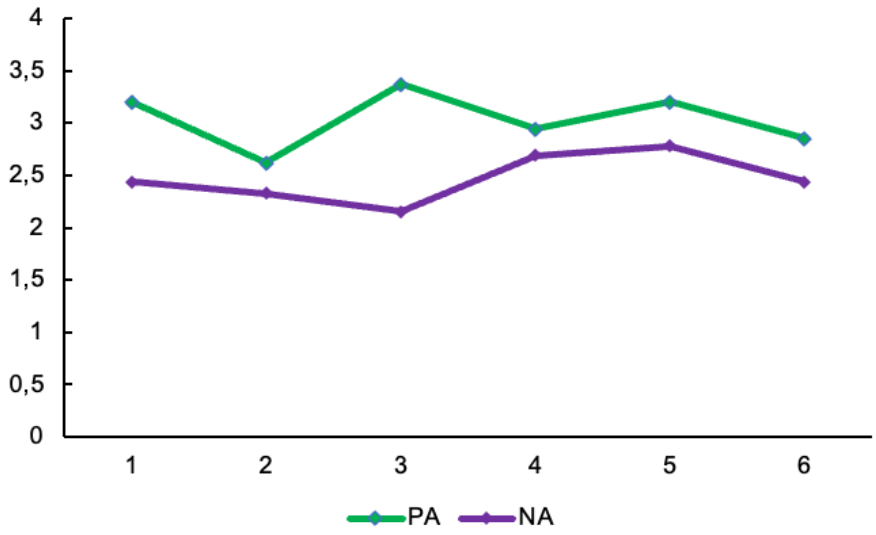

Supplement: Supplementary file 1 — Supplementary Material 1 [file 41598_2026_40521_MOESM1_ESM.pdf]
